# Emerging liver organoid platforms and technologies

**DOI:** 10.1186/s13619-021-00089-1

**Published:** 2021-08-03

**Authors:** Do Thuy Uyen Ha Lam, Yock Young Dan, Yun-Shen Chan, Huck-Hui Ng

**Affiliations:** 1 Laboratory of precision disease therapeutics, Genome Institute of Singapore, 60 Biopolis Street, Singapore, 138672 Singapore; 2 Department of Medicine, Yong Loo Lin School of Medicine, National University of Singapore, 10 Medical Dr, Singapore, 117597 Singapore; 3 Division of Gastroenterology and Hepatology, University Medicine Cluster, National University Hospital, 5 Lower Kent Ridge Road, Singapore, 119074 Singapore; 4 Bioland Laboratory (Guangzhou Regenerative Medicine and Health Guangdong Laboratory), Guangzhou, 510005 China; 5 Department of Biochemistry, National University of Singapore, Singapore, 117559 Singapore; 6 NUS Graduate School for Integrative Sciences and Engineering, National University of Singapore, 28 Medical Drive, Singapore, 117456 Singapore; 7 Department of Biological Sciences, National University of Singapore, 14 Science Drive 4, Singapore, 117597 Singapore

**Keywords:** Liver, Organoids, Stem cells, Disease models, Regenerative therapy

## Abstract

Building human organs in a dish has been a long term goal of researchers in pursue of physiologically relevant models of human disease and for replacement of worn out and diseased organs. The liver has been an organ of interest for its central role in regulating body homeostasis as well as drug metabolism. An accurate liver replica should contain the multiple cell types found in the organ and these cells should be spatially organized to resemble tissue structures. More importantly, the in vitro model should recapitulate cellular and tissue level functions. Progress in cell culture techniques and bioengineering approaches have greatly accelerated the development of advance 3-dimensional (3D) cellular models commonly referred to as liver organoids. These 3D models described range from single to multiple cell type containing cultures with diverse applications from establishing patient-specific liver cells to modeling of chronic liver diseases and regenerative therapy. Each organoid platform is advantageous for specific applications and presents its own limitations. This review aims to provide a comprehensive summary of major liver organoid platforms and technologies developed for diverse applications.

## Background

The liver is one of the largest organs in the human body and is responsible for a number of functions (Trefts et al. 2017). The liver is the primary organ that regulates nutrient absorption and metabolism, neutralizes ingested toxic compounds and produces close to 50% of the circulating proteins in the body. To maintain such diverse roles, the liver is composed of a variety of cell types that are spatially organized to form distinct functional structures. The most abundant parenchymal cell type in the liver is the hepatocytes which are responsible for majority of the organ functions. The hepatocytes are tightly organized in lobules where cells in varying zones express different sets of functional genes in response to signals in the micro-environment (Gebhardt and Matz-Soja 2014; Halpern et al. 2017). The hepatocytes are also highly polarized where the apical surface is exposed to the blood plasma and the lateral surface interacts to form a continuous canaliculus for the secretion of bile (Treyer and Müsch 2013). The bile canaliculus is responsible for the transport of secreted bile towards the bile ducts formed by the cholangiocytes. The parenchymal cholangiocytes are responsible for constructing the illustrious network of ducts across the entire liver organ and consolidates the bile secretions towards the gastrointestinal system (Boyer 2013). There are multiple non-parenchymal cell types including the hepatic stellate cells (HSC), kupffer cells (KC) and liver sinusoidal endothelial cells (LSEC) that contribute to the liver function and more importantly, to protect and maintain the tissue integrity (Malik et al. 2002). Recent single cell atlas of the human liver shows that potentially more than 10 different cell types (Aizarani et al. 2019) interact and form tissue structures that are fundamental to maintain the organ integrity and function in response to injuries and diseases.

The liver is subjected to various metabolic, viral and xenobiotic insults that induce liver injuries while performing daily routine functions (Greuter and Shah 2016). Liver injury involves a dynamic interplay between the different liver cell types that results in the gradual loss of hepatocytes and extensive remodeling of the liver architecture, which eventually reduces the liver functional capacity (Giannelli et al. 2003). In most liver diseases and drug induced liver injuries (DILI), the insults begin in the hepatocytes and the diseased cells trigger an injury response that activates other cell types such as the KC and HSC. Prolonged injury induces the formation of insoluble fibrotic scars in the organ that disrupt organ integrity and also induces systemic injury such as portal hypertension (Malhi and Gores 2008). Liver diseases and injuries of various etiologies likely also involves the interplay of different cell types. Hence, to fully understand how each of the liver disease manifests and consumes the organ, we require more sophisticated in vitro human model systems that are capable of recapitulating many of these cellular interactions.

There have been numerous strategies to recreate the human liver architecture in the laboratory and such 3D cell culture are commonly referred to as liver “organoids”. The advantages of organoid culture, consisting of single or multiple liver cell types, include 1) reconstruction of liver tissue structures in vitro (Ramli et al. 2020; Collin de l’Hortet et al. 2019), 2) recapitulation of interactions between different cell types in the organ during development, homeostasis or disease progression (Ouchi et al. 2019; Collin de l’Hortet et al. 2019), 3) propagation of primary cells from the liver and tumor (Huch et al. 2013; Hu et al. 2018), 4) Improvement of physiological functions of the liver cells through the mimicry of tissue niche (Rennert et al. 2015) and 5) enhancement of vascularization in the organoids in vitro and in vivo (Takebe et al. 2013). Due to these advantages, organoids systems are increasingly adopted in all areas of liver research including modeling of human liver development and diseases, regenerative therapy, and drug response and toxicology studies. The methods to generate these organoids range from simple culture techniques using cell culture matrices such as collagen and matrigel to provide the 3D environment for the cells to interact and self-organize, to more complex bioengineering approaches that employ de-cellurized organ scaffolds or bio-printing technology to precisely control and achieve cell layering to generate 3D structure. Each method employs cells ranging from primary cells derived from tissue, somatic cells differentiated from stem cells and immortalized cell lines. Selection among these cells are largely dependent on functional maturity of cells (primary cells), ability of cells to survive under harsh printing or culture conditions (immortalized cell lines), and scalability of cell culture for constructing large organoids or for high-throughput screens (stem cell-derived somatic cells). Some organoid systems employ more than one type of cell source for co-culture.

Liver organoids of different sizes, shapes and cell compositions have been reported and could be broadly classified into several categories: 1) Stem cell and progenitor organoids which consist of proliferative cells that can be expanded in scale and differentiated to liver cell types; 2) Pluripotent stem cell (PSC) derived liver organoids that include hepatocyte or cholangiocyte organoids, hepatobiliary organoids composed of both parenchymal cell types, and multi-cellular liver organoid that consists of the parenchymal cells and the non-parenchymal mesenchymal, endothelial or immune cells; 3) Bio-engineered liver organoids that engaged the use of cell layering and patterning techniques, together with bio-fabrication and bio-printing technologies to create 3D cultures of single or multiple liver cell types. Each liver organoid system presents its unique advantage such as the ease of adoption (technical and resource limitations), scalability and animal-free components for therapeutics application, and cellular and structural maturity for modeling different aspects of liver disease and injury. In the following sections, we will review major studies in each category of liver organoids.

## Main text

### Stem cell and progenitor liver organoids

#### Tissue-derived biliary and hepatic organoids

**Fig. 1:**
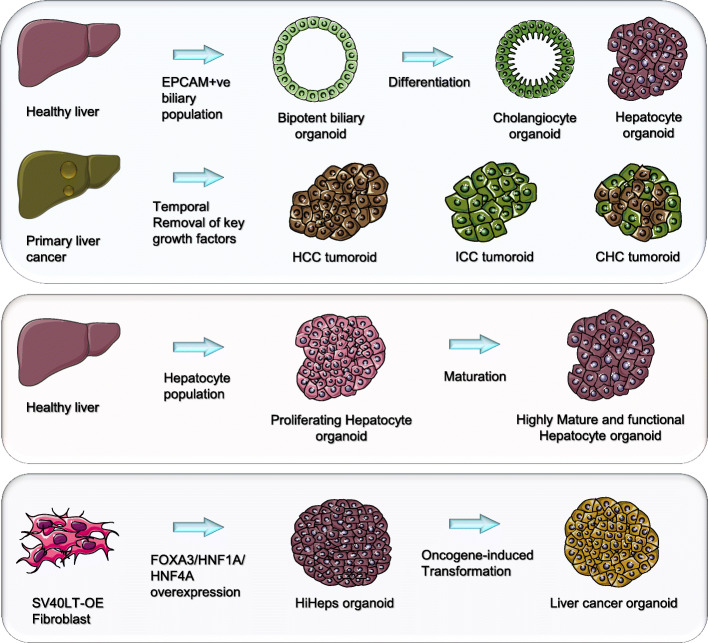
Stem cell and progenitor liver organoids. Top panel: Proliferating bipotent biliary organoids isolated from patient liver tissue can be maintained in culture as an expandable cell source and differentiated into functional cholangiocytes or hepatocytes. This approach has been employed to derived tumoroids from the three major liver cancer subtypes. Middle panel: Using similar approach with additional hepatocyte supporting signaling factors, proliferating hepatocytes can also be derived from liver tissues. These proliferating hepatocytes can be further matured into highly functional hepatocytes. (Bottom panel): Transdifferentiation of fibroblast to liver organoids. Fibroblast over-expressing (OE) SV40 large T antigen (SV40LT) is transdifferentiated into human-induced hepatocyte organoids (HiHeps), using a combination of FOXA3, HNF1A and HNF4A. Introduction of various HCC oncogenes transform the HiHeps into liver cancer organoids

The ability to expand patient-derived primary liver cells has been of great interest and a major challenge for basic and translational research. The realization of this goal will provide sufficient patient-matched materials for molecular research to better understand disease manifestation. In recent years, organoid technology pioneered by the Clever’s group has been harnessed to successfully expand patient-derived material (Sato et al. 2009). Using a cocktail of growth factors and small molecules, the group was able to derive LGR5^+^ adult stem cells from small intestine tissues, which expand as spherical cystic structures embedded in a matrix. This study demonstrates the feasibility of establishing adult stem cells (ASCs) from progenitors in human organs and expanding these patient-derived tissues as mini-organ like structures in culture. Using a similar approach, Clever’s group reported the first liver organoid derived from mouse and human adult liver tissue (Huch et al. 2013; Huch et al. 2015) (Fig. 1A). These human adult liver organoids were highly expandable in culture for up to 6 months and maintained a stable genome (Huch et al. 2015). The group also showed that these cells likely originate from the EpCAM-enriched biliary cells within the liver tissue. In contrast to the intestinal organoids, these biliary organoids expanded largely as populations of homogenous epithelial stem cells that require further differentiation to generate functional cell types. Nonetheless, these biliary stem cell organoids are shown to be bipotent and could differentiate to generate both parenchymal cell types. Importantly organoids generated from A1-Antitrypsin (A1AT) deficiency and Alagille syndrome patients recapitulated the in vivo disease pathology. This landmark study demonstrates for the first time, the potential to generate large quantities of patient-specific liver cells for various research and translational applications.

While the biliary organoids could potentially generate large quantities of hepatocytes in culture, these organoids of biliary origin appear to be recalcitrant in differentiating to hepatocytes in vitro or when engrafted into mice (Hu et al. 2018). This limits the applications of the biliary organoid platform as hepatocytes are the major cell type of interest for both research and translational applications. The liver is a highly regenerative organ where populations of hepatocytes have been shown to repopulate the injured liver (Gadd et al. 2020). Harnessing this proliferative potential of hepatocytes, two independent groups developed culture conditions to derive expandable hepatocyte organoids (Fig. 1A). Clever’s group further supplemented cytokines that promote hepatocyte cell fate into the biliary organoid culture media (Huch et al. 2015) to formulate new culture condition that enabled the expansion of mouse and human hepatocytes in similar 3D culture approach (Hu et al. 2018). In a parallel effort, Nusse’s group showed that TNFα, an inflammatory cytokine enriched during liver injury, could induce mouse hepatocyte proliferation in culture (Peng et al. 2018). In contrast to the biliary organoids that maintain a spherical cystic structure, the human hepatocyte organoids distinctly grew as a compact “bunch of grapes” (Hu et al. 2018). The hepatocyte organoids exhibit liver functions comparable to primary human hepatocytes and interact to form MRP2^+^ bile canaliculi. In contrast to the biliary organoids, the hepatocyte organoids efficiently repopulated the mouse liver when engrafted. Transcriptomic analysis suggests that these cells closely resemble hepatocytes in proliferation upon partial hepatectomy (Hu et al. 2018). However, in contrast to the biliary organoids, human hepatocyte organoids was only efficiently derived from hepatocytes of fetal origin. Isolation efficiency from pediatric and adult liver reduces to 1% and organoids exhibit a much-limited expansion capacity (only up to 2–3 months). These short-comings in derivation efficacy and expansion capacity limit the potential use of this technology in generating patient-specific liver disease model and large scale expansion for therapeutic applications.

#### Liver cancer organoids

One of the major achievements with the abovementioned organoid culture platform is the generation of patient-derived tumor organoid model for various cancer indication including breast cancer (Sachs et al. 2018), prostate cancer (Gao et al. 2014), bladder cancer (Lee et al. 2018), glioblastoma (Hubert et al. 2016) and multiple cancer of the gastrointestinal system (Broutier et al. 2017; Sato et al. 2011; Vlachogiannis et al. 2018) including colon (van de Wetering et al. 2015) and pancreas (Boj et al. 2015). The organoid platform has immense potential as human tumor surrogate model for precision medicine due to the high derivation efficacy of the methodology for specific cancers, and the organoid’s ability to recapitulate the endogenous tumor architecture and expand in culture (Friedman et al. 2015). Huch’s group demonstrated that the biliary organoid technology can be optimized for the isolation of primary human liver cancer cells (Broutier et al. 2017) (Fig. 1A). Remarkably, the platform can isolate cancer organoids from all three common liver tumor subtypes, namely hepatocellular carcinoma (HCC), intrahepatic-cholangiocarcinoma (ICC) and Hepato-Cholangiocarcinoma. The group further demonstrated the use of these expandable patient-specific cancer “tumoroids” for screening therapeutic options. In light of the stem cell base culture media employed, it will be of interest to further investigated whether these tumor organoid models are enriched for tumor initiating cells (TIC) or cancer stem cells (CSC). While the technology is promising as a precision medicine platform for patient-specific therapeutic discovery in HCC, the low derivation efficacy for liver tumors with well-differentiated lesions and contamination of non-cancer epithelial organoids remain a major hurdle for wide-adoption of this technology.

In an independent study, Clever’s group show that the biliary organoid can be genetically engineered to create cholangiocarcinoma models (Artegiani et al. 2019). Using the CRISPR-Cas9 system, the group introduced common cholangiocarcinoma mutations in *NF1*, *PTEN*, *SMAD4* and *TP53* genes into the biliary stem cells to induce oncogenic transformation. Using this cholangiocarcinoma organoid model, the group demonstrated the tumor suppressor role of BAP1, where genetically modified BAP1^−/−^ biliary organoids acquire malignant features in vivo. Taking a novel approach to develop HCC and ICC cancer organoid models, Sun et al. make use of functional human induced hepatic organoids (hiheps) generated from fibroblast immortalized with SV40 large T antigen, which inactivates p53 and Rb proteins (Sun et al. 2019) (Fig. 1). Subsequent overexpression of the Myc oncogene is sufficient to induce oncogenic transformation in the hiheps which form tumors when engrafted. Using this model, the group further elucidated the early molecular and biogenesis events during Myc-induced HCC formation, and demonstrated potential ICC transformation from hiheps overexpressing common reported ICC oncogenic factors such as RAS. Importantly, both studies demonstrated the immense potential of the organoid systems in creating in vitro human liver cancer models that enable the study of molecular and cellular events during oncogenic transformation. These organoid models provided a much needed resource in the field of liver cancer study to understand pre-neoplastic events, and identify potential biomarkers for detection of early liver cancer development.

While these stem cell and progenitor organoid platforms enabled the derivation of patient specific liver cells and cancer organoids, the organoids described thus far are largely progenitor in nature and differentiated organoids consist of a single cell type and lacks most liver-specific structures. As adult stem cells are largely lineage-restricted, it remains unclear whether these cells alone can be used to generate organoids containing more liver cell types.

### Pluripotent stem cell-derived liver organoids

**Fig. 2:**
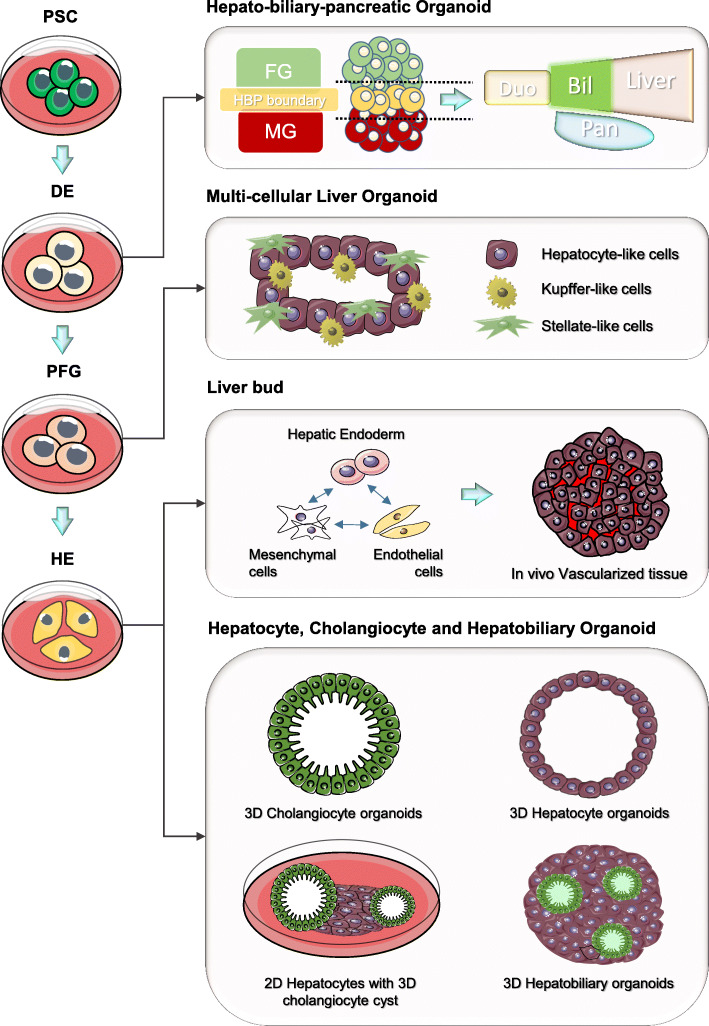
Pluripotent stem cell (PSC) derived liver organoids. PSCs are provided with signalling cues to differentiate along the endoderm lineage into cells resembling the definitive endoderm (DE), posterior foregut (PFG) and hepatic endoderm (HE). These intermediate endoderm lineage progenitors are utilized to generate various liver organoids. The DE can use to generate the midgut (MG) and foregut (FG) spheroids. The MG and FG co-culture induces hepato-biliary-pancreatic (HBP) organogenesis at the boundary to generate the HBP organoids containing cell and structure resembling the duodenum (Duo), pancreas (Pan) and liver that are interconnected by the biliary (Bil) structure. PFG can be employed to generate multi-cellular liver organoids containing both parenchymal (hepatocytes) and non-parenchymal (HSCs and Kupffer cells) cells. The HE can be co-culture with mesenchymal stem cells and HUVEC to produce liver buds that further mature into vascularized tissues when transplanted in mice. Multiple groups have also further differentiated the HE to generate liver organoids that contain only cholangiocytes or hepatocytes, or hepatobiliary organoids containing both liver parenchymal cell types in different configurations

One of the most widely adopted approach to recreate an organ-like structure in a dish is to mimic organogenesis during embryo development. This strategy exploits the prior knowledge obtained from both developmental studies in model organisms as well as differentiation experiments using PSCs. The self-renewing capacity and developmental potential of PSCs promoted the use of these cells for deriving organoids resembling various human organs (Kim et al. 2020). In comparison to the tissue-derived organoids, PSC-derived organoids in general consist of more cell types that self-organized to form complex structures (Fig. 2). As organoid systems are rapidly developed and used for modeling diseases and developmental processes in various fields, the organoids generated (of the same organ) are diverse in cellular composition and structure which becomes challenging to define. Subsequently, a set of criteria to defined PSC-derived organoids was described (Lancaster and Knoblich 2014): In essence, an organoid contains multiple organ-specific cell types that interact to form structure resembling the organ and is capable of performing organ-specific functions. In this section, we will review major PSC studies which generated liver organoids that fulfilled most if not all of these criteria.

#### Liver bud

One of the earliest PSC-derived liver organoids described is the liver bud from Taniguchi’s group (Takebe et al. 2013), which employs a co-culturing approach to assemble different liver cell types. The initial liver bud described consists of PSC-derived hepatic endoderm cells, human umbilical vein endothelial cells (HUVECs), and human mesenchymal stem cells (MSCs) which upon transplantation into the mice, generated functional and vascularized liver tissue (Fig. 2). This approach recreates the niche environment of embryonic liver development where the hepatic endoderm is enveloped by mesenchymal and endothelial cells in the septum transversum. The different cell types interacted on a soft matrix and self-organize to form a 3D structure that resembles the E10.5-E11.5 liver bud in mouse embryo (Takebe et al. 2013). The group achieved a fully PSC-derived (single iPSC donor) liver bud in a following study (Takebe et al. 2017), which also described an improvised platform that enables a 100-fold increase in liver bud production capacity. While the initial liver bud holds great promise for regenerative medicine, the immature state limits application as human culture model of the organ system. In an attempt to use this liver bud for in vitro disease modeling, Taniguchi’s group further devised an in vitro maturation strategy to generate functional liver cells for modeling hepatitis B infection (Nie et al. 2018). However, the differentiated liver bud consist of largely hepatocyte-like cells and lack other functional cell types or liver organ structure compared to the liver bud matured in the mice. It remains to be determined whether the liver bud could a suitable platform for generating mature and functional liver organoids in culture.

#### Hepatobiliary organoids

A common strategy for generating organoids from PSC involves the step-wise lineage commitment of the cells through various embryonic stages and towards the organ of interest. This strategy was employed by multiple groups to induced PSC to form liver organoids through intermediate endoderm stages including the definitive endoderm, posterior foregut and the hepatic endoderm/hepatoblast which give rise to the liver organ (Collin de l’Hortet et al. 2019; Ouchi et al. 2019; Ramli et al. 2020; Wu et al. 2019) (Fig. 2). Key advantages of this strategy include the establishment of intermediate progenitor cells that enable expansion in cell number and more importantly, the recapitulation of the entire liver organ formation that could reveal insights into early human embryonic liver development.

Using this step-wise PSC differentiation strategy, Wu et al. generated the first hepatobiliary organoid that contains both parenchymal cell types (Wu et al. 2019). The group devised a three-step approach that includes the maintenance of a small population of mesenchymal cells in the first two phases. The mesenchymal cells mimic the role of the septum transversum in development, similar to the liver bud approach. The hepatobiliary organoid contains a monolayer of hepatocytes with cholangiocyte cysts forming in adjacent. These hepatobiliary organoids can give rise to hepatic tissue consisting of both parenchymal cell types when transplanted into the mouse splenic capsule. While the authors demonstrated the function of individual parenchymal cells, there is a lack of investigation in the functional and structural interactions between the two cell types. In a parallel effort, Ramli et al. designed a similar stepwise approach to generate a hepatobiliary organoid in 3D suspension culture (Ramli et al. 2020). The liver organoid is composed of a dense spherical hepatocyte core with cholangiocyte cysts of various sizes at the periphery. In this study, the authors showed that the organoids form a continuous network of bile canaliculi that connects the hepatocytes with the cholangiocyte cysts, recapitulating the bile transport network in the liver tissue. The study further demonstrated the use of this structural feature to model both drug-induced and disease-induced cholestasis. Of note, the protocol described enables the production of organoids in high-throughput format and achieved significant consistency in size and cellular composition. This broadens the potential application of these hepatobiliary organoids including high content screens. Interestingly, in both studies, the cholangiocytes are effectively derived under hepatocyte inducing conditions. Unravelling this differentiation propensity of the hepatoblast within the organoids will further shed light to the early lineage specification events during human embryonic liver development.

#### Multi-cellular liver organoids

While the hepatobiliary organoids capture the interaction between the 2 major cell types in the liver, it lacks other non-parenchymal cell types (mesenchymal, immune and endothelial cells) which are important for organ homeostasis and tissue response during disease and injury. Ouchi et al. described one of the first protocol to create a PSC-derived liver organoid that consist of both parenchymal and non-parenchymal cell types to model steatohepatitis (Ouchi et al. 2019). In comparison to the hepatobiliary organoid protocols, the authors describe a relatively efficient method (20 days) to derive a hepatic organoid containing hepatocyte-like cells, Stellate-like cells and Kupffer-like cells. Further treatment of the organoids with fatty acids for 5 days was sufficient to induce inflammatory and fibrosis responses. Of note, the authors were able to demonstrate that the fatty acid treated liver organoids developed increased stiffness with atomic force microscopy, which resembles liver stiffness observed in patients with steatohepatitis. The study also demonstrated the key advantage of PSC-derived organoids where genetic diseases can be modelled using patient-derived induced PSC (iPSC). While structural changes during disease progression were captured in this study, there is a lack of detail report of structural features in the liver organ. This is likely due to the immature state of the cells derived under a relatively short timeframe. Further optimization of organoid incubation period and the culture conditions may be necessary for the cell types to mature and self-organize to form tissue-like structures.

The organoid studies described thus far aim to create mature liver organ for investigating liver function, model disease and for regenerative applications. A potential use of the PSC-derived organoid system is to investigate the process of liver organogenesis. Till date, much of our knowledge on early embryonic liver development was uncovered using small rodent models, and species-specific differences may confound the translation of these findings. To model the early gut morphogenesis events that generated the embryonic liver, Koike et al. established a co-culture system of SOX2^+^ anterior and CDX2^+^ posterior gut spheroids that further give rise to the hepatic-biliary-pancreatic (HBP) organoid (Koike et al. 2019). Remarkably, the interaction of the midgut and foregut spheroids initiated the formation of the HBP without the need for additional signalling cues. Continuous culture for up to 90 days shows that the HBP domain can give rise to hepatic and pancreatic tissues with connecting ducts that resemble the E10.5 mouse HBP domain explants. Using this system, the authors were able to show that HES1 is also important for human HBP formation, as observed in knockout mice. This report validates the potential use of organoids platform to understand and dissect molecular events regulating human embryonic liver morphogenesis.

Till date, many other groups have also described protocols that generated hepatic organoids from PSC which consist largely of either stem cells or single liver cell type. Many of the studies focused on generating cholangiocytes or hepatocytes in 3D and mounting evidence suggest that the cells generated as organoids are more functional compared to cells in 2D monolayer (Kim et al. 2020; Zhang et al. 2018a). These organoids have been employed for all aspects of liver studies including investigation of drug-induced liver injury (Sampaziotis et al. 2015), viral hepatitis (Nie et al. 2018), liver toxicity (Shinozawa et al. 2020) and alcoholic liver injury (Wang et al. 2019). Table 1 provides a list of major liver organoid studies and summarizes the features and approaches employed to generate the organoids.

**Table 1:** Major liver organized studies continued

Studies	Type of organoid	Approach	Unique features and applications
**Huch et al. 2013, 2015**	Biliary stem cell organoid	- - Isolation and expansion of EPCAM+ biliary cells from liver biopsy - 3D matrigel embedded culture	- Stable expansion of patient-specific adult liver biliary stem cells - Bipotent biliary stem cells can generate functional hepatocytes and cholangiocytes in culture and form functional hepatocytes when engrafted in mice (low efficacy)
**Takebe et al. 2013**	Liver bud	- Co-culture of iPSC-derived hepatic cells, HUVECs and MSCs - Cells co-culture in 2D on matrigel forms 3D liver bud	- Self-organization of multiple cell types in vitro into a immature liver bud (lacks liver function) - Liver bud develops into functional and vascularized liver tissue when engrafted into immuno-deficient mice - Engrafted liver bud rescues genetic liver failure in mice
**Ogawa et al. 2015**	Cholangiocyte-like cell (CLC) organoids	- Step wise differentiation of hiPSC through bipotent hepatoblast - 3D matrigel embedded culture	- Cholangiocytes exhibit mature biliary markers and structures such as apical sodium-dependent bile acid transporter, secretin receptor, cilia and cystic fibrosis transmembrane conductance regulator (CFTR) - In vitro modelling of cystic fibrosis with patient derived iPSC
**Sampaziotis et al (2015, 2017)**	CLC organoids	- Step wise differentiation of hiPSC through bipotent hepatoblast - 3D matrigel embedded culture	- CLC is highly functional and utilized for validating polycystic drugs - Organoids were used to model cystic fibrosis - Engrafted CLC enable treatment of common bile duct (CBD) disorders
**Broutier et al. 2017**	Cancer organoids	- Isolation of cancer cells from liver tumor tissue with modified media and protocol (Huch et al. 2015) - 3D matrigel embedded culture	- Derivation of the cancer organoids (tumoroids) from major liver cancer subtypes including Hepatocellular Carcinoma (HCC), Intrahepatic Cholangiocarcinoma (ICC) and Hepato-Cholangiocarcinoma - Tumoroids retain histological features of patient-derived tumor tissue after long term culture, preserve genetic alteration from original tumor and show metastatic potential - Tumoroids were used for drug screening to identify potential patient specific therapy
**Takebe et al. 2017**	Liver bud	- Co-culture of single donor iPSC-derived hepatic endoderm, endothelial and mesenchymal cells - Engineered platform for large scale production	- Single donor iPSC-derived hepatic, endothelial and mesenchymal cells to generate patient specific liver buds - Mechanized engineering platform for robust large scale production of liver bud - Liver rescue experiments achieved in over 100 mice to demonstrate potential for regenerative therapy
**Hu et al. 2018**	Hepatocyte organoid	- Isolation and expansion of hepatocytes from mouse liver and fetal human liver - 3D Matrigel embedded culture	- Hepatocyte organoid exhibit high engraftment efficiency in mice - The proliferating hepatocyte organoid resembles proliferating hepatocyte in liver with partial hepatectomy - Hepatocyte organoid can be further matured in culture and exhibit major liver metabolic functions and forms bile canaliculi
**Peng et al. 2018**	Hepatocyte organoid	- Expansion of isolated hepatocytes from mouse liver using pro-inflammatory cytokine TNFα - 3D matrigel embedded culture	- Hepatocyte proliferation induced using inflammatory signals enriched in injured liver - Hepatocyte organoids exhibit major liver functions and shows high engraftment capacity
**Nie et al. 2018**	Liver bud	- Isolation of endothelial and mesenchymal cells from a single umbilical cord donor for liver bud organoid formation	- Reduced liver bud size and maturation *in vitro* - Differentiated liver bud consist mainly of hepatocytes and used for modeling HBV infection
**Zhang et al. 2018;** **Fu et al. 2019**	Hepatocyte organoid	- Expansion of isolated hepatocytes as a 2D proliferating hepatocyte progenitor - Hepatocyte progenitor mature in 3D to form hepatocyte organoids	- Hepatocyte progenitor platform enabled large scale expansion of isolated primary adult human hepatocytes - Hepatocyte progenitor mature into functional hepatocyte in vitro and in vivo. Cells demonstrate comparable engraftment efficiency compared to primary human hepatocytes - Matured 3D hepatocyte organoids can be used for modelling HBV infection and reactivation in vitro
**Vyas et al. 2018**	Hepatobiliary organoid	- Isolated human fetal liver progenitor cells (FLPC) are seeded and maintained in decellularized liver (ferret) disc	- FLPC repopulated decellularized liver disc scaffold and differentiate to give rise to hepatobiliary organoid containing functionally mature AFP-ve hepatocytes and bile duct cells of different maturity - Hepatobiliary organoid was used to investigate role of notch signaling in bile duct formation
**Wu et al. 2019**	Hepatobiliary organoid	- Stepwise 2D differentiation of iPSC into hepatoblast and hepatobiliary cells - Maintenance of a minimal mesenchymal population for hepatobiliary formation	- Cells differentiated in 2D culture generated a monolayer of hepatocytes with 3D biliary cysts - Addition of cholesterol based reagent maintains hepatobiliary cells in culture and promotes maturity - Transplanted organoids give rise to tissue containing both bile ducts and hepatocytes
**Ouchi et al. 2019**	Multi-cellular organoid	- Stepwise differentiation of iPSC into liver organoid using an intermediate foregut spheroid progenitor - 3D matrigel embedded culture	- Single cell analysis shows that organoid contains both parenchymal and non-parenchymal cell types of the liver - Liver organoid treated with free fatty acid exhibit inflammatory response, undergo fibrogenesis which results in increase stiffness of organoids
**Collin de l’Hortet et al. 2019**	Multi-cellular organoid	- Co-culture of iPSC derive hepatic cells with human microvascular endothelial cells, human mesenchymal cells, fibroblasts and macrophages in a decellularized rat liver scaffold	- Large organoid measuring centimetres in size - Modeling of Sirt1 depletion induced NASH under fatty acids enriched media - NASH Model is capable of recapitulating steatosis, inflammation and ballooning phenotype - Incorporation of media perfusion system enable NASH phenotype penetration in the core of large organoid.
**Wang et al. 2019**	Bipotent hepatic progenitor	- Stepwise differentiation of PSC into bipotent hepatic progenitor organoids - 3D matrigel embedded culture	- Highly expandable progenitor organoids that can generate hepatocytes or cholangiocytes in vitro and in vivo - Co-culture of fetal-liver mesenchymal cells improves hepatocyte functions in organoid and enable modelling of alcohol-induced liver injury
**Sun et al. 2019**	Hepatocyte and Cancer organoid	- SV40LT expressing fibroblast are transdifferentiated into hepatocytes (hiHeps) and cultured as 3D organoids in suspension - Overexpression of HCC and ICC oncogenes to induce neoplastic transformation	- hiHep aggregates show polarity and exhibit better hepatocyte function than 2D cultured hiHep - Introduction of Myc oncogene or ICC enriched mutations induces HCC and ICC transformation respectively. The transformed organoids form tumor in mice, that recapitulate features of patient derived tissue. - Platform enables recapitulation of molecular and biogenesis events during neoplastic transformation
**Artegiani et al. 2019**	Biliary Cancer organoid	- Cholangiocarcinoma associated genetic mutations are introduced into tissue derived biliary organoid to induce oncogenic transformation	- Step-wise transformation of biliary organoids into cholangiocarcinoma organoids enable the investigation of tumor suppressor function of BAP1 - In vitro transformation platform enables investigation into cellular and molecular changes during neoplastic transformation
**Mun et al. 2019; Akbari et al. 2019**	Biliary stem cell organoid	- Isolation and expansion of EPCAM+ bipotent progenitor from pluripotent stem cells - 3D matrigel embedded culture	- Bipotent Progenitor derived from PSC is similar to the biliary stem cell organoid platform (Huch et al. 2015) - Bipotent progenitor differentiates to hepatocytes in 3D, which exhibit liver functions and demonstrates regenerative and inflammatory responses. - iPSC platform enable modeling of genetic disease with patient derived fibroblast
**Koike et al. 2019**	hepatic-biliary-pancreatic (HBP) endoderm organoid	- Co-culture of PSC-derived midgut and foregut spheroids. - Spheroid boundary further develops into HBP - 3D matrigel embedded culture	- Spontaneous induction of hepatic-biliary-pancreatic domain formation and outgrowth to form bile duct linked hepatic and pancreatic tissues - Validation of the role of HES1 in human liver and pancreas organogenesis.
**Ramli et al. 2020**	Hepatobiliary organoid	- Stepwise differentiation of PSC in 2D and 3D culture - 3D suspension culture	- Hepatobiliary organoid with a dense hepatocyte core and cystic cholangiocytes in the periphery - Functional bile canaliculi network connecting both cell types observed in the organoid. - Modelling of drug and disease induced cholestasis.
**Shinozawa et al. 2020**	Hepatocyte organoid	- Stepwise differentiation of PSC in matrigel droplets - Generation of an expandable foregut progenitor intermediate	- Spherical hepatocyte organoid containing a lumen that enable modelling of bile transportation - High-throughput screening with organoids identified cholestasis inducing toxins

### Bio-engineered liver organoids

**Fig. 3:**
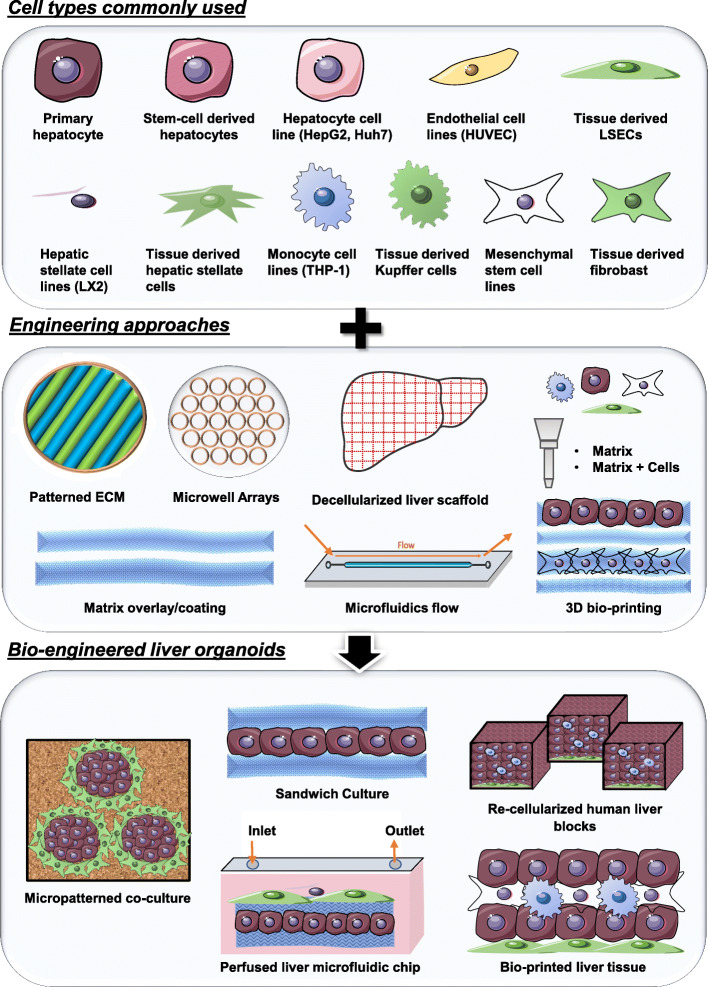
Engineering approaches to create liver organoids. Different parenchymal and non-parenchymal liver cell types have been employed with a variety of engineering tools to create bio-engineered liver organoids. Cell sources range from primary cells derived from tissues to immortalized cell lines and cells differentiated from expandable stem cells. Non-parenchymal endothelial, mesenchymal and Kupffer cells are the common stroma and immune liver cell types employed to recapitulate the niche environment of the liver tissue. Cells are seeded with precision in controlled environment using one or more common bio-engineering platforms and approaches such as patterned ECM, microwell arrays, matrix overlays, microfluidics platform and 3D bio-printing. Common bioengineered organoids generated using the various cell types with different engineering approaches are depicted

Before the advent of organoid platforms described so far, there have been numerous bio-engineering approaches to reconstruct the liver organ in vitro. While it is beyond the scope of this review to cover all these engineering methods, we aim to give a concise summary of some of the major strategies employed including: 1) Micro-manipulation of cell culture vessels and matrices, 2) microfluidics culture system for fluid exchange and 3) three dimensional scaffolds and bio-printing of matrices and cells. Bio-engineers employ one or more of these strategies as well as different sources of liver cell types (primary cells, immortalized cell lines and stem cell derived hepatic cells) to create different bioengineered 3D liver culture system (Fig. 3).

#### Micro-manipulation of cell culture vessels and matrices

A common engineering approach to redesign the physical microenvironment for cell culture is the use of fabricated micropatterned surfaces. A common form of this system comprises of cell culture dish surfaces imprinted with a defined array of extracellular matrices (ECM) that directs cellular adhesion (Khetani and Bhatia 2008; Khetani et al. 2013; Ware et al. 2015). The customized printing of single or multiple ECM enables unique patterns of cell seeding to achieve different configurations on the culture dish. One of the most versatile micropatterned liver co-culture (MPCC) system generated, comes from Bhatia’s group where the micropatterned disc of primary hepatocytes, endothelial cells and fibroblast have been employed to model diseases such as viral Hepatitis B (Shlomai et al. 2014) and C (Ploss et al. 2010), plasmodium infection (March et al. 2013), and toxicological studies (Khetani et al. 2013). More recently, MPCC-derived liver organoids also show great potential for regenerative therapy in pre-clinical models (Stevens et al. 2017). An intriguing alternative strategy to pattern cells without reliance on pre-fabricated ECM surfaces involves the use of dielectrophoresis (DEP) (Ho et al. 2013). The use of electrodes embedded in fluidics chip enabled high precision patterning of the HepG2 and HUVEC cells onto centimeter size lobule structures. This alternative approach opens up new avenues to create large liver lobule structures with higher cell patterning precision.

Another common form of engineered hepatic organoid culture that relies on ECM manipulation is the sandwich culture. In this approach, hepatic cells are grown in between layers of matrix which often includes collagen or Matrigel (Bi et al. 2006; Chatterjee et al. 2014; Oorts et al. 2016). In addition to performing basic functions of hepatocytes such as albumin secretion and CYP activities, hepatocytes grown in sandwich culture are shown to exhibit polarity due to different interacting surfaces (De Bruyn et al. 2013). A variant of this sandwich culture is the layer-by-layer cell coating technique reported by Akashi’s group (Sasaki et al. 2017). In this study, three hepatic cell types were coated with fibronectin and gelatin, and seeded in layers to form tissue-like structures. By manipulating the number of layers of each cell type, the authors demonstrated the importance of the dermal fibroblast in promoting vascularization by the endothelial cells and the cells correspondingly form functional vascularized liver tissue when engrafted into nude mice.

#### Microfluidics culture system: liver organ-on-a-chip

Both micropatterned surface and sandwich culture methods described can be employed on micro-chip system that includes fluidics to create a perfusion platform. These microfluidics platforms are commonly referred to as ‘Organ-on-a-chip’ that aims to capture the physiology of the organ such as tissue-tissue interface and importantly the vascular system. Most microfluidics culture incorporate a flow system with an inlet and outlet to enable the continuous exchange of substances including culture media and therapeutic compounds. Most systems employ at least two liver cell types configured in layers (Du et al. 2017; Kang et al. 2015; Rennert et al. 2015; Tomlinson et al. 2019), tube-like layers (Ahmed et al. 2017), or with each cell types in isolated chambers (Lee et al. 2013). The perfused cultures/co-culture reportedly enhances formation and maintenance of liver structure such as bile canaliculi and cells exhibit superior liver functions such as albumin secretion, urea synthesis and CYP activities. These advantages were well demonstrated in the liver organoid system generated by Rennert et al., where the group recreated the liver sinusoid with opposing fluid system mimicking the bile and vascular flow (Rennert et al. 2015). The team utilized a microfluidic system where the vascular layer (endothelial and Macrophage) and hepatic layer (hepatocytes and HSCs) are separated by a semi-porous disc and separate fluidic systems were used for layer-specific media in opposing flow directions. The findings show that the cells in the liver organoid under the perfusion system exhibit structural features such as formation of microvillus and enhanced metabolic functions. With the incorporation of sensors in the microfluidic chip, the group was also able to monitor in-situ oxygen levels that could be a surrogate to assess micro-environment in real-time. This fluidic and oxygen monitoring system holds the potential for establishing nutrient and oxygen gradients in the liver organoids which are essential for recapitulation of liver zonation.

#### Three dimensional scaffolds and bio-printing

Scaffolding is a widely-adopted method to create tissue-like cultures as it provides a “skeletal structure” of the organ for cells to adhere and organize. One of the conventional ways to create scaffold is the use of decellularized animal or human liver. This method is highly advantageous for reconstructing the human liver organ as the liver scaffold contains physiologically relevant ECM constitution and architecture. The decellularized liver scaffold is subsequently repopulated with human liver cell types (Collin de l’Hortet et al. 2019; Hussein et al. 2016; Mazza et al. 2015; Vyas et al. 2018).

Collin de l’Hortet et al. generated one of the largest reported liver organoid with a combination of bioengineering approaches to create a physiological model of Non-alcoholic steatohepatitis (NASH) (Collin de l’Hortet et al. 2019). The authors differentiated hepatocytes from human iPSC and embedded these cells with human microvascular endothelial cells, human mesenchymal cells, human fibroblasts, and human macrophages into a decellularized rat liver scaffold. A key challenge to many liver organoid protocols reported is the balance of the size of the organoid and their maintenance in culture. Static cultures are unfavourable for large organoids as the core will be devoid of nutrients and oxygen. By incorporating perfusion and cell infusion pump systems, the organoid generated in this study measured up to a few centimetres in size and was reportedly maintained up to 10 days. More importantly, parallel comparison with static culture shows that the flow system is important for the recapitulation of the NASH pathology in the entire organoid. Impressively, the disease organoid sections exhibit close physiological resemblance to corresponding human liver disease tissues. The system remains to be refined to include HSCs for modelling fibrosis and it is also unclear whether the endothelial cells form functional vascular structures under the perfusion system. It will also be interesting to investigate if a functional vascular system is able to introduce liver zonation in these large centimeter size organoids. Hepatocytes distributed across different zones between the portal triad and central vein exhibit characteristic and functional differences in response to the nutrient and oxygen gradient (Kietzmann 2017). Recreating an organoid with hepatocytes resembling those in all three zones will reveal whether the zonation, which are often disrupted during disease manifestation, plays a role in the disease progression.

Challenges for the wide-adoption of the decellurized organ scaffold technology in labs include the technicality involved in decellularizing the liver and manipulating the size of the scaffold for various applications. 3D bio-printing using bio-compatible matrices such as galactosylated alginate or chitosan-gelatin gels, emerges as an alternative to create such scaffolds (Gong et al. 2014; Arai et al. 2017; Lewis et al. 2018). Common scaffold structures printed include simple sandwich culture configurations (Arai et al. 2017), more complex rectangular laden structures where porosity could be manipulated to enhance hepatocyte specific functions (Lewis et al. 2018) and hierarchical channel networks which aim to mimic the liver vascular system (Gong et al. 2014). Progressively, researchers are now incubating liver cells into common matrices such as fibronectin, gelatin, alginate (Nikolova and Chavali 2019) and lyophilized tissue ECM scaffold (Skardal et al. 2015) to generate bio-ink for direct 3D printing. The technology is still at its infancy and most studies aim to maintain cell viability and physiology under the harsh bio-printing environment (Kizawa et al. 2017; Skardal et al. 2015; Leva et al. 2018). Cells can be printed directly onto a platform (Leva et al. 2018) or cultured as spheroids and mixed with bioink before printing (Skardal et al. 2015). In another case, these spheroids are skewered together on 3-by-3 pole structures to create more complex 3D culture (Kizawa et al. 2017). Thus far, researchers have been able to bio-print immortalized cell lines and primary hepatocytes including co-culture of the different cell types. Cells are usually printed in sequential layers to achieve tissue scale depth. Most of these cells can be maintained in culture longer than monolayer primary hepatocytes, express liver-specific markers, as well as exhibit basic liver functions such as urea synthesis and albumin production (Faulkner-Jones et al. 2015; Jeon et al. 2017; Kang et al. 2017; Kim et al. 2017; Kim et al. 2018; Lee et al. 2017; Lee et al. 2016; Wang et al. 2006). One of the first bio-printed organoids in multiple transwell system from Presnell’s group demonstrates potential broad applications for such bio-printed liver organoids (Justin B. Robbins 2013; Nguyen et al. 2016). These organoids consist of a core of primary hepatocytes with non-parenchymal primary HSCs and HUVECs printed at the periphery. Printed hepatocytes can survive and maintain functions up to 28 days in culture and currently utilized for DILI testing (Nguyen et al. 2016) and modeling liver diseases (Norona et al. 2016).

Overall, bioengineering approaches have proven to be advantageous for creating 3D liver culture as the use of different bio-materials coupled with precision placement technologies enable better spatial arrangement of cells to generate different organ structure. Different configurations are employed to create models of different sizes and constitutions to fit diverse applications (Fig. 3). Engineering approach also introduces the much-needed flow system required for mimicking the vascular system which is also essential for building larger and more complex liver tissue in vitro. On the other hand, challenges for adopting these approaches include limited availability of technologies and materials, specialized training required for operator and harsh culture conditions which limits cell type used.

### Perspective: liver organoids for disease modeling and therapeutics

#### Disease modeling using liver organoids

The modeling of human disease phenotype and progression is a key application for human in vitro models. Organoid technology brought about a breakthrough for the research field as the model greatly facilitated the study of multiple cell type interactions and tissue-level changes during disease manifestation and progression. Each of the organoid platform described so far have been employed to model different liver diseases and each present its unique advantages.

The stem cell and progenitor organoid platform provided a solution to the major challenge of establishing and expanding patient-specific liver cells for in vitro molecular and drug response studies. The technology is highly advantageous for creating genotype specific human models of liver diseases including A1-Antitrypsin Deficiency (Gómez-Mariano et al. 2020; Huch et al. 2015), Alagille Syndrome (Huch et al. 2015) and Primary Sclerosing Cholangitis (Soroka et al. 2019). While iPSC-derived organoids also enables the generation of similar patient-specific liver disease models (Akbari et al. 2019; Guan et al. 2017; Nie et al. 2018; Ouchi et al. 2019; Sampaziotis et al. 2015), the tissue-derived stem cell organoid system is advantageous for generating liver cancer models (Broutier et al. 2017; Li et al. 2019; Nuciforo et al. 2018). On the other hand, the hepatobiliary and multi-cellular liver organoids have proven to be useful for the modeling of disease phenotype that requires the interplay between different parenchymal and non-parenchymal liver cell types (Collin de l’Hortet et al. 2019; Ouchi et al. 2019) and involves tissue structural changes (Ramli et al. 2020). These models are suitable for the modeling of complex diseases such as NAFLD where the pathological process involves different cell types as the disease progress. The key advantage of the bio-engineered organoids in modeling liver disease and injury lies in the precision achieved with various engineering approaches. This includes tight control of cell arrangement, density and microenvironment, achieved during cell culture vessel manufacturing, cell seeding, and integration of real-time monitoring system. Hence, bioengineered organoids have been widely employed for drug response and drug toxicity studies (Hussein et al. 2016; Ware et al. 2015; Zhu and Li 2017), that require highly controlled culture environment for quantitative assays.

While each describe platform has been advantageous for liver disease modeling in various frontiers, a major expectation for liver organoid is in the modeling of disease phenotypes that remains a challenge to conventional single cell type, monolayer culture systems. This includes the modeling of liver inflammation and fibrogenesis which are converging phenotype for all major liver diseases, and involves a coordinated response of multiple parenchymal and non-parenchymal liver cells. These phenotypes include the many structural changes observed in the liver tissue architecture that have yet to be captured in vitro.

#### Liver inflammation, fibrogenesis and structural changes

The immune cells such as macrophages and T cells play an indispensable role in mediating inflammatory response (Robinson et al. 2016). Critical events of liver inflammation include the recruitment and migration of immune cells through the sinusoid, and the cellular crosstalk via a plethora of secreted cytokines and chemokines and cell-cell interaction. Arguably, these critical events are excellent targets for therapeutic interventions and the lack of human models impede their discovery. The modeling of such complex liver inflammatory response was partly recapitulated in Collin de l’Hortet et al’s report (Collin de l’Hortet et al. 2019). The bio-engineered organoid was treated with free fatty acids cocktail to recapitulate NAFLD development. The liver organoid achieved tissue-like features that enabled assessment of NAFLD Activity Score (NAS). This includes the full spectrum of inflammation severity (score of 0–3) which are quantified by the number of macrophage loci detected. Fibrogenesis, which often develops after inflammatory responses in the liver, involves the formation and accumulation of insoluble fibers that consist largely of ECM proteins secreted by the activated HSCs. The HSCs are located in the space of disse, a fine gap between the hepatocytes and the endothelial cells and play a role in lipid and vitamin A storage in the quiescent state. A multitude of different secretory factors from almost all cell types (hepatocytes, immune cells, endothelial cells, platelets and biliary epithelial cells) in the liver regulates HSC activation in the disease or injured liver (Foglia et al. 2019). Hence, an accurate model of liver fibrogenesis will need to capture these molecular cross-talks governing HSCs activation (Lee and Friedman 2011). Capturing the secretome and physical interaction between these cell types under healthy and disease conditions would be essential for unraveling molecular events that maintain HSC quiescent and myofibroblast transformation. The PSC-derived liver organoid described by Ouchi et al. consists of multiple hepatic-like cells, resembling the majority of the abovementioned cell types important for fibrogenesis (Ouchi et al. 2019). Using these liver organoids, the authors was able to recapitulate increase tissue stiffness during NAFLD induced fibrogenesis.

While both organoid studies capture aspects of liver inflammation and fibrosis response, the models still lack several key features to fully model these complex events. One of the major challenges in organoid culture system is the creation of culture conditions that could support a variety of cell types in their physiological or disease states. Almost all organoid systems described so far do not contain other important immune cell types such as the adaptive immune T cells that are reported to play an important role in liver inflammation (Her et al. 2020). Few studies so far have also shown compelling evidence of quiescent HSC culture for modeling fibrogenesis (Coll et al. 2018) including the above described organoid studies. Most fibrogenesis modeling studies likely fail to capture this quiescent to activation transition of HSCs, and mainly measures the induction of collagen/ECM secretion by partially or fully activated HSCs under the culture conditions. The co-culture of HSCs and adaptive immune cells within the liver organoids could be a critical challenge as these cells are easily activated/influenced under harsh culture conditions and overexposure to multiple signaling factors that are enriched in organoid culture media. Another major challenge for many of the organoids described is the lack of spatial organization of the different liver cell types. Most liver organoid studies primarily focus on achieving co-culture of multiple liver cell types where cells remain functional and are able to recapitulate disease phenotype upon stimulation. There is a lack of organoids studies that aim to reconstruct organ level structure such as the liver lobule, sinusoids and the bile canaliculi network. Reconstructing these liver architectures is crucial for investigating important events for liver inflammatory responses such as immune cells recruitment from the sinusoids. The HSCs are physiologically situated between these 2 layers and such orientation could also be important for regulation of the cell state. We have witness the importance of liver cell types interaction and spatial organization, for vascularization and functional maturation of the organoids (Takebe et al. 2013; Wu et al. 2019; Rennert et al. 2015). It is likely that the diverse cell type interaction and spatial arrangement will also be essential for accurate modeling of disease phenotypes.

The bile canaliculi network is a fundamental liver organ structure that functionally connects the parenchymal cell types. Obstruction of this intra-hepatic bile transport system, commonly known as cholestasis, accounts for majority of DILI (Padda et al. 2011). DILI is an important part of drug development and a leading cause of attrition of preclinical and clinical drugs due to liver failure (Roth and Lee 2017). To this end, Ramli et al. reported a hepatobiliary organoid model that exhibits a functional bile transport network (Ramli et al. 2020). The authors demonstrated the utility of this organoid in modeling DILI induced by Troglitazone. Live-imaging of the drug-treated organoids show that the cholestasis-inducing drug significantly reduced molecule transport into the bile canaliculus network. In addition, the organoid treated with FFA also recapitulated micro-cholestasis that occurs in NAFLD patients of different disease stages (Segovia-Miranda et al. 2019). Moving forward, there would be a need for more organoid systems that are capable of capturing other structural architecture of the liver to mimic important remodeling events of the liver tissue observed in different diseases and injuries (Almeda-Valdés et al. 2017; Dara et al. 2017; Fu and Li 2017; Fukui 2017; Katoonizadeh 2017; Thuyle et al. 2017; Xia et al. 2017). For instance, fibrogenesis events begin at the peri-portal and peri-sinusoidal regions of the liver lobule and progress to form bridging septas in later stages (Giannelli et al. 2003). Such site specific initiation and progression events should be modeled with organoids that can recapitulate the periportal and sinusoidal liver structures. On the other hand, this would likely be a significant challenge as many PSC-derived organoid approaches do not have tight control over the liver cells interaction during differentiation process, and are highly dependent on the differentiating cells to self-organize to form various structures.

#### Liver organoids for regenerative medicine

In the past 40 years, orthotopic liver transplantation (OLT) is the most widely adopted and ideal therapeutic solution for end-stage liver failure and is projected to continue as the gold standard (Alwahsh et al. 2018). Despite the good outcome of OLT, limited donor availability remains the major hurdle for the treatment. This has prompted significant interest to develop alternative sources of hepatic tissue to rescue the failed liver. The discovery of stem cell-derived hepatic cells (Hu et al. 2018; Huch et al. 2015; Ouchi et al. 2019; Ramli et al. 2020; Takebe et al. 2013; Takebe et al. 2017; Vyas et al. 2018) and improvement in cell maturation techniques especially in 3D format (Chen et al. 2018), has prompt hepatologist to explore the potential of using in vitro derived hepatic cells for regenerative therapeutics (Forbes et al. 2015). One of the simplest form of cell therapy is to transfuse primary hepatocytes. This method has been used to treat acute liver failure due to genetic disorders (Dhawan et al. 2004; Horslen et al. 2003; Ribes-Koninckx et al. 2012; Sokal et al. 2003). However, this method has similar availability issue as OLT and isolated hepatocytes rapidly de-differentiates in culture. Due to this inherent issue, stem cell-derived hepatic cells cultured in 3D format with supporting stromal cells, that extend the cell survival and function prior to transplantation, are currently explored (Messina et al. 2020). The liver bud from Taniguchi (Nie et al. 2018; Takebe et al. 2013; Takebe et al. 2017) and MPCC organoids from Bhatia’s group (Stevens et al. 2017) described earlier are examples of organoid cultures with high engraftment capacity for regenerative therapies. The common theme employed by each system is the introduction of supporting stromal cells and endothelial cells which greatly enhanced vascularization of the transplanted cell mass. This enabled the long-term survival, expansion and functionality of the engrafted cell mass. To note, the system described by Taniguchi’s group has achieved remarkable scalability (Takebe et al. 2017) and also demonstrated the possibility to use different cell types derived from a single iPSC clone (Zhang et al. 2018b). This system has now been validated in larger porcine models for clinical safety with portal vein transplantation procedures and shows great promise for human clinical trials in the near future (Tsuchida et al. 2020).

An ultimate goal of the organoid platform for regenerative medicine would be the generation of sizeable bioartificial liver for OLT. These liver organoids should be centimeters in size, consist of essential liver cell types including the parenchymal and stroma cells and perform major liver functions in culture. Importantly, the cells should be spatially organized in lobules and form liver tissue structures such as the canaliculi and blood vessels to facilitate integration into the host liver tissue. In essence, the liver organoid is expected to achieve near physiological liver function and structural features in culture to increase the chances that the bioartificial liver integrates and functions post-transplantation. Current liver organoid model fall short of these expectations due to one or more of these factors: 1) The absence of functional vascular system to deliver nutrients and air, and facilitate metabolite and waste exchange for centimeter size organoids, 2) various cell types lacks spatial organization and poorly resemble the liver tissue architecture, 3) immature cell types with poor liver functions and 4) dependence on matrices with animal components such as Matrigel for 3D culture. Importantly, any approach to reconstruct the organ likely need to tackle multiple of these challenges at the same time. For instance, in constructing a functional vascular system, the required spatial organization of LSEC in the sinusoid, as well as mature functional LSC features such as membrane fenestration need to be achieved in parallel. Similarly, hepatocytes need to be spatially aligned, exhibit polarity to form functional transport networks, and perform various metabolic and detoxification functions. Overcoming these challenges likely require combinations of approaches such as the use of engineering platforms to introduce media flow and achieve precision placement of cells, and stem cell-based differentiation approach to generate sufficient quantities of various liver cell types from a single donor. The potential of such combinatorial approach was evident in the study by Yanagi et al. where the group employed 3D bio-printing technology to create liver bud culture (Yanagi et al. 2017). In comparison to the above-mentioned liver bud culture method, the use of 3D bio-printing of the 3 major cell types enabled the “stitching” of hundreds of liver bud in vitro in specific geometry. The sizable liver tissue was maintained and matured through the introduction of a media circulation flow.

#### Organoids-on-a-chip

We are witnessing an increasing trend in the integration of bio-engineering approaches and organoid culture techniques to create more physiological in vitro organ models (Achberger et al. 2019; Nikolaev et al. 2020). Nikolaev et al. generated adult tissue derived intestinal organoids on a microfluidic platform that could be maintained for over a month without the need for subculturing. The circulation system, achieved with the microfluidics set up, provided fresh media and removal of shedding dead cells. Remarkably, the introduction of the flow system also improved the organoid differentiation capacity where rare intestinal cells such as the microfold cells and enteroendocrine cells were found and enriched in the intestinal organoid-on-a-chip system as compared to the traditional static culture. Importantly, the prolong organoid maintenance and accessibility of the organoid lumen in this culture system, greatly facilitated the successful in vitro human modeling of the full cycle of *Cryptosporidium parvum* parasitic activities after infection. Employing similar organoid-on-a-chip system for precision spatial placement of cells and introduction of vascular system, Achberger et al. generated a Retina-on-a-chip platform that enabled the co-culture of Retinal organoids (RO) with retinal pigment epithelial (RPE) cells. Employing a combination of hydrogels, microfluidics compartments, flow system and PSC-derived RPE and RO, the team generated a polarized layer of RPE that recapitulated its function as a blood-retinal barrier between the retinal organoids and circulation system. The group demonstrated that this setup significantly improved the inner and outer retinal segment formation and integrity, while enabling a direct interplay between the RPE and photoreceptors to recapitulate events such as the phagocytosis of the photoreceptor outer segment by the RPE. Overall, these two studies highlighted the advantages of organoid cultures on microfluidic system which includes the introduction of a functional vascular structure, greater control of organoid size and growth, and precision placements of cells and organoids to recapitulate physiological structures. In the near future, we would likely witness the development of similar liver organoids-on-a-chip which will provide solutions to the highlighted challenges for applications in liver disease modelling and regenerative medicine.

## Conclusions

We have witnessed a rapid growth in interest to generate and utilize organoid platforms in various fields of liver studies. Multiple groups have employed different strategies to recreate organ-resembling 3D cultures of different shape, size and composition. This have partly resulted in a lack of clarity on organoid definitions and poses challenge for scientific communication, where this review hopes to resolve for liver organoids. Of note, a consensus on the definition and nomenclature for hepatic, pancreatic, and biliary (HPB) organoids was recently established by the HPB Organoid Consortium (formed by over 60 experts from 16 countries) (Marsee et al. 2021). This consensus will provide clarity on types of organoids available and achieve consistency in organoid description in future publications. Many of these organoids are “tailored” towards specific studies and applications, and researchers now have a repertoire of liver organoid systems that they could adopt. This trend would likely continue given the challenges to generate organoid that fully recapitulate the liver organ structure and functions remain. The precision control of cell spatial organization, maintenance of cell state and function in harsh and complex signaling environment, and introduction of functional vascular system in vitro remain key issues for liver organoids generated for both disease modeling and regenerative therapy. Employing combinations of stem cell and bio-engineering approaches described in this review to create the next generation liver organoids, will be the key strategy to overcome these hurdles.

## Data Availability

Not applicable.
